# Implementation strategies to promote measurement-based care in schools: evidence from mental health experts across the USA

**DOI:** 10.1186/s43058-022-00319-w

**Published:** 2022-06-21

**Authors:** Elizabeth H. Connors, Aaron R. Lyon, Kaylyn Garcia, Corianna E. Sichel, Sharon Hoover, Mark D. Weist, Jacob K. Tebes

**Affiliations:** 1 Department of Psychiatry, Yale University, 389 Whitney Avenue, Office 106, New Haven, CT 06511 USA; 2 Department of Psychiatry and Behavioral Sciences, University of Washington, 6200 NE 74th Street, Suite 100, Seattle, WA 98115 USA; 3 Department of Psychology, University of South Carolina, 1512 Pendelton Street, Columbia, SC 29208 USA; 4 Division of Child/Adolescent Psychiatry, Department of Psychiatry, Columbia University, 1051 Riverside Drive, New York, NY 10032 USA; 5 Department of Psychiatry, University of Maryland, 737 West Lombard Street, 4th Floor, Baltimore, MD 21201 USA

**Keywords:** Implementation strategy selection, Measurement-based care, School mental health treatment

## Abstract

**Background:**

Despite an established taxonomy of implementation strategies, minimal guidance exists for how to select and tailor strategies to specific practices and contexts. We employed a replicable method to obtain stakeholder perceptions of the most feasible and important implementation strategies to increase mental health providers’ use of measurement-based care (MBC) in schools. MBC is the routine use of patient-reported progress measures throughout treatment to inform patient-centered, data-driven treatment adjustments.

**Methods:**

A national sample of 52 school mental health providers and researchers completed two rounds of modified Delphi surveys to rate the relevance, importance, and feasibility of 33 implementation strategies identified for school settings. Strategies were reduced and definitions refined using a multimethod approach. Final importance and feasibility ratings were plotted on “go-zone” graphs and compared across providers and researchers to identify top-rated strategies.

**Results:**

The initial 33 strategies were rated as “relevant” or “relevant with changes” to MBC in schools. Importance and feasibility ratings were high overall for both survey rounds; on a scale of 1 to 5, importance ratings (3.61–4.48) were higher than feasibility ratings (2.55–4.06) on average. Survey 1 responses resulted in a reduced, refined set of 21 strategies, and six were rated most important and feasible on Survey 2: (1) assess for readiness and identify barriers and facilitators; (2) identify and prepare champions; (3) develop a usable implementation plan; (4) offer a provider-informed menu of free, brief measures; (5) develop and provide access to training materials; and (6) make implementation easier by removing burdensome documentation tasks. Provider and researcher ratings were not significantly different, with a few exceptions: providers reported higher feasibility and importance of removing burdensome paperwork than researchers, providers reported higher feasibility of train-the trainer approaches than researchers, and researchers reported higher importance of monitoring fidelity than providers.

**Conclusions:**

The education sector is the most common setting for child and adolescent mental health service delivery in the USA. Effective MBC implementation in schools has the potential to elevate the quality of care received by many children, adolescents, and their families. This empirically derived, targeted list of six implementation strategies offers potential efficiencies for future testing of MBC implementation in schools.

**Supplementary Information:**

The online version contains supplementary material available at 10.1186/s43058-022-00319-w.

Contributions to the literature

- Delivering mental health treatment services in schools improves access for children and adolescents, but school context-specific implementation strategies are needed to increase availability of best practices in this setting.
- Measurement-based care (MBC) is a scalable, evidence-based clinical practice that can be added to nearly any school-based mental health treatment approach, but school systems, administrators, and providers need targeted supports to increase MBC implementation.
- Important and feasible strategies to implement MBC in schools focus on training and supporting mental health providers plus addressing systemic barriers such as burdensome documentation.
- Stakeholders in this study agreed on the feasibility and importance of various implementation strategies for MBC with some exceptions; providers emphasized the importance and feasibility of removing burdensome paperwork and researchers emphasized the importance of fidelity monitoring.
- We used an established, stakeholder-engaged method to systematically select and tailor implementation strategies that can be replicated for other practices and settings.

## Background

Evidence-based practices (EBPs) continue to proliferate in child and adolescent mental health treatment, many of which are developed under controlled conditions in university clinics and healthcare settings [1]. However, intervention evidence is limited by the client populations and settings where the evidence was originally derived [2], often making it necessary to adapt the intervention to fit a particular setting [3, 4]. In addition, there are numerous barriers to successful EBP implementation in real-world mental health settings where children and their families are likely to receive care. Implementation barriers exist in both the outer setting (e.g., patient needs and resources) and the inner setting (e.g., organizational culture, leadership engagement), as well as involving individual characteristics and implementation processes unique to each intervention, intervention level, population, and service setting [3].

Desired implementation outcomes are more likely when implementation strategies are selected for and tailored to 1) specific patient populations, 2) care delivery systems and practices, and 3) local barriers and facilitators, often referred to as “determinants of practice” [5, 6]. Implementation strategies are single- or multiple-component approaches aimed at increasing adoption, implementation, and sustainment of EBPs in routine care [7]. Despite an established taxonomy of 73 implementation strategies, minimal guidance exists for how to select, integrate, and tailor these strategies to specific services and contexts [8, 9]. Proposed methods include concept mapping, group model building, conjoint analysis, and intervention mapping [10]. Yet, each method has limitations, such as requiring advanced methodological consultation, complex modeling that may overwhelm stakeholders, and/or use of proprietary software [10].

There are few examples of how to select strategies prospectively based on implementation science research and stakeholder knowledge of contextual factors [5]. This study replicates one established systematic method (the use of modified Delphi surveys) to select implementation strategies for a given EBP (measurement-based care) in the most common mental health service delivery setting for children and adolescents (schools) [10]. Delphi surveys are a pragmatic approach [10] that can be used when implementation strategy lists are established and thus stakeholders can rate existing strategies, propose new ones, and recommend changes in strategy definitions or applications. Stakeholder ratings of *importance* and *feasibility* have been used in numerous studies to assess which strategies are most actionable and applicable for a given implementation initiative to maximize success [11–16]. The actual effectiveness of these strategies on implementation, service, and client outcomes is an empirical question to be evaluated once they are applied [7].

## Measurement-based care in mental health service delivery

Measurement-based care (MBC) is the routine collection and use of client data throughout treatment, including initial screening and assessment, problem definition and analysis, finalizing treatment objectives and intervention tactics, and monitoring treatment progress collaboratively with the client to inform treatment adjustments [17]. MBC is a critical component of an evidence-based practice orientation to mental health treatment [18]. There is strong evidence supporting MBC in settings other than schools. For instance, systematic reviews show better and faster goal attainment and symptom reduction with MBC as compared to usual care; effect sizes range from 0.28 to 0.70 [19–21]. Larger effect sizes of 0.49 to 0.70 are attributable to MBC with feedback, particularly feedback provided to both the patient and providers, or when clinical support tools are provided [21, 22]. Recent Cochrane reviews underscore the importance of including studies where measures are used to adjust the treatment plan [23, 24], indicating that patient outcomes associated with MBC are likely a result of the real-time, client-centered, data-driven adjustments made to interventions provided.

Despite the promise of MBC to improve mental health service quality, use of MBC in practice is minimal. Fewer than 20% of providers report collecting progress measures at least monthly [25, 26]. Barriers to MBC implementation in behavioral health care have been well-documented at the individual patient, provider, organizational, and system levels [27].

## School mental health treatment services

Schools are the most common setting for children to receive mental health treatment, particularly for families who face barriers to accessing care in traditional clinic- or hospital-based settings [28–32]. However, the extent to which school mental health treatment services are grounded in EBPs is largely unknown [33, 34]. EBPs implemented in schools have potentially broad reach [35, 36] and school-based EBP implementation allows for adaptation to local culture and contexts that is scalable across communities and states [37, 38].

### Implementation considerations in schools

Selecting and tailoring implementation strategies to practice and context has been found to optimize implementation feasibility and, ultimately, effectiveness outcomes [39, 40]. Yet, results are mixed, suggesting that tailoring may need to occur continuously throughout implementation [41]. Schools are also a unique setting for mental health treatment services, so implementation strategies defined for other behavioral healthcare delivery settings are unlikely to fit perfectly for schools without attention to strategic selection and tailoring. Indeed, implementing new practices in educational settings requires careful attention to school organizational factors, such as principal leadership, education policies at state and federal levels, a heterogenous mental health workforce, requirements and constraints related to professional development and ongoing coaching, and logistics as basic as the school calendar [42]. Other studies point to the importance of flexible treatment delivery and intentional family engagement efforts to facilitate EBP implementation and outcomes [43].

### MBC implementation in schools

Barriers to MBC implementation in schools have some similarities with other more traditional behavioral health care settings, such as providers reporting limited time to administer measures. However, some barriers are more salient in the school context, such as difficulty reaching parents, limited access to measures, and lack of administrative or technical resources for scoring measures [44]. Although scientifically-rigorous applications of MBC in schools are new, an individualized approach to monitoring student progress and outcomes has been emphasized and studied in schools for decades [45, 46]. There are some published demonstrations of standardized, patient-reported outcome measures being implemented in school mental health systems [47, 48], as well as examples of psychosocial progress monitoring in schools as part of high-quality, comprehensive school mental health systems [49]. Moreover, MBC is consistent with schools’ emphasis on Response to Intervention, which is using student progress data to prevent and remediate academic and behavioral difficulties [50] and accountability requests for school-based providers to demonstrate outcomes [51]. Recent studies have highlighted case examples of an MBC approach in schools, from assessment tool selection to measurement processes and the role of feedback to the student and family [51, 52]. Yet, there still remains a substantial gap in the literature regarding implementation strategies best suited to MBC implementation when child mental health treatment services are provided on school grounds instead of a more traditional clinic or hospital setting.

## Current study

The current study identifies feasible and important implementation strategies to increase school mental health provider use of MBC. This work builds on an initial list of 70+ implementation strategies that have been codified for general use [9, 53], and a recent extension to identify top strategies relevant to and important for implementing evidence-based practices in school settings [13, 54]. We focused specifically on selecting strategies for MBC in schools using prior Delphi survey methods. We collected importance and feasibility ratings for implementation strategies as well as operational definitions and recommendations for practical application in schools [9, 13, 53, 54]. Our objective was to obtain stakeholder perceptions of the most feasible and important implementation strategies for MBC as rated by provider and researcher stakeholders with expertise in school mental health treatment.

## Methods

### Participants

Study participants (*N* = 52) were drawn from a national sample of school mental health stakeholders: (1) providers with experience delivering and/or supervising mental health interventions in schools (*N* = 31); and [2] researchers with experience partnering with schools or districts to implement EBPs (*N* = 21). Providers were sampled from the National School Mental Health Census and researchers were sampled from two established lists of researchers with relevant expertise (see procedures for details). All participants were US-based in one or more than 23 states (AZ, AR, CA, CO, CT, FL, GA, IL, IN, LA, MD, MA, MI, MN, NE, NH, NC, OH, OR, PA, TX, VA, and WA). Table 1 shows demographic, professional, and urbanicity characteristics of participants.

**Table 1:** Demographic and professional characteristics of stakeholder participants, *N* = 52

Characteristic	Total sample		Providers		Researchers	
	*n*	%	*n*	%	*n*	%
Age						
21–30 years	1	2	1	3	–	–
31–40 years	11	21	9	29	2	10
41–50 years	21	40	15	48	6	29
51–60 years	13	25	6	19	7	33
61 and over	6	12	–	–	6	29
Gender						
Female	27	52	26	84	11	52
Male	25	48	5	16	10	48
Race/ethnicity						
Asian or Asian American	1	2	–	–	1	5
Caucasian or White	42	80	23	74	19	91
Hispanic (Spanish descent)	4	8	4	13	–	–
Latino/a/x (South or Central American descent)	2	3	2	7	–	–
Multiracial	3	6	2	7	1	5
Field of training^a^						
Clinical or counseling psychology	12	23	6	19	6	29
School psychology	16	31	10	32	6	29
Social work	11	21	10	32	1	5
Special education	5	10	–	–	5	24
Multiple fields	1	2	–	–	1	5
Professional counseling	6	12	6	19	–	–
Substance use/addiction counseling	1	2	1	3	–	–
Other (school counseling, rehabilitation counseling services)	11	21	9	29	2	10
Degree^a^						
PhD	25	48	4	13	21	100
EdD	1	2	1	3	–	–
PsyD	1	2	1	3	–	–
LCSW	4	8	4	13	–	–
LGSW	1	2	1	3	–	–
LCPC	2	3	2	7	–	–
LMFT	1	2	1	3	–	–
BA/BS	5	10	5	16	–	–
MA/MS	15	29	15	48	–	–
Other (MEd, LMSW)	10	19	10	32	–	–
Urbanicity^ba^						
Metro	38	72	20	65	18	86
Nonmetro	24	46	11	35	13	62

*Note. N* = 52 *(n* = 31 providers and *n* = 21 researchers)

^a^ Participants selected all that apply for these characteristics so they add to greater than 100%

^b^Urbanicity for SMH researchers refers to their partnerships, not where they work personally

Providers identified as school psychologists (*N* = 6, 19%), school social workers (*N* = 5, 16%), or school counselors (*N* = 5, 16%). Other provider roles included being a school psychology supervisor (*N* = 2, 7%), director of related services/special education/student support (*N* = 2, 7%), counselor (community- or hospital-employed; *N* = 1, 3%), mental health agency administrator (*N* = 1, 3%), or other positions (*N* = 9, 29%). School providers were based in 18 states representing all regions of the USA, and researchers in 14 states and had worked with school partners in 43 states, the District of Columbia, Guam, the US Virgin Islands, and other US territories. Most providers indicated they had current or past experiences delivering (*N* = 30, 97%) and/or supervising (*N* = 20, 65%) mental health treatment services in schools. Demographic and professional characteristics and urbanicity of the *N* = 31 participating providers displayed in Table 1 were not significantly different from those *N* = 53 providers recruited who completed the prescreening survey, based on chi-square tests (Awad M, Connors E: Promoting measurement-based care in school mental health through practice-specific supervision, submitted). These details were not available for individuals who completed the School Mental Health Profile generally.

Researchers had experience conducting research about child and adolescent mental health, conducting research in partnership with schools/districts, training school-based personnel, and providing consultation or technical assistance to schools/districts. Most researchers had current or past experience training graduate students about working in or with schools (*N* = 20, 95%), providing mental healthcare in schools (*N* = 16, 77%), supervising direct mental healthcare in schools (*N* = 13, 62%), and serving as an educator (*N* = 11, 52%). Researchers represented various age groups, fields of training, and urbanicity across the USA. Although gender identity (56% female) and degree (100% PhD) appear similar to researchers in our datasets who were not invited to participate, we did not have detailed self-reported characteristics of non-participating researchers to conduct statistical comparisons. Results from study participants are likely generalizable to stakeholders of similar demographics, professional expertise, and geographic location. There was a 94% retention rate of participants for Survey 2 (*N* = 49; *N* = 30 providers and *N* = 19 researchers).

### Procedures

Systematic sampling procedures that drew on nationally representative databases for school-based providers and researchers were used to identify the study sample. Providers were selected through stratified random sampling from the National School Mental Health Census, a nationally representative survey of school and district mental health teams’ services and data usage. Inclusion criteria, confirmed by self-report on a prescreening survey, was holding a position as a school mental health provider or clinical supervisor with experience delivering or supervising school-based psychotherapy, in which MBC would be used (e.g., school social worker). Census data with individuals meeting this inclusion criteria were stratified based on rural-urbanicity continuum codes (metropolitan vs. non-metropolitan) and geographic representation. Prospective participants were randomly selected with replacement until a target sample of at least 30 school mental health providers was achieved. We monitored the sample for approximate distributions in the USA for (1) metropolitan and non-metropolitan/rural urbanicity; and [2] geographic location. Using this approach, we oversampled for non-metropolitan/rural providers toward the end of recruitment to ensure adequate representation.

We recruited 211 school mental health providers; after a response rate of 25% (*N* = 53) for a prescreening survey, four were ineligible due to never being a clinician or clinical supervisor (*N* = 3) or community provider not working in a school (*N* = 1). Of the *N* = 49 eligible participants invited to complete Survey 1, a final sample of 31 providers participated. Eligible recruits who did not participate had nonworking emails (*N* = 24), did not respond to our recruitment request (*N* = 106), or declined (*N* = 28). Providers received up to three reminder emails over the course of 3 weeks to respond to the study invitation to consent and start Survey 1.

Researchers were selected using purposive sampling from two sources, which were (1) Implementation Research Institute fellows who applied to and were selected for implementation science training through a competitive process and reviewed for school mental health expertise [55]; and (2) a list of 138 school mental health researchers maintained by the National Center for School Mental Health with active peer-reviewed publications and/or grants on topics pertaining to school mental health and wellbeing. This latter group of researchers were part of an invitation-only annual national meeting and pre-reviewed for their scholarship and impact on the field, adjusted for career stage, by a planning committee team comprised of national school mental health scholars. Inclusion criteria were (1) expertise with mental health program or practice development, effectiveness testing, and/or implementation research; (2) experience partnering directly with schools; and (3) Associate Professor or Professor at their institution, which resulted in *N* = 56 eligible researchers. Next, advanced expertise implementing mental health programs or practices in schools was coded on a 4-point scale (3 = “optimal,” 2 = “good,” 1 = “okay’, and 0 = “unable to assess”) by three senior school mental health researchers with extensive experience in evidence-based practice implementation in schools. Ratings were averaged for each researcher and then recruits were invited with replacement from the highest ratings downwards until a sample size of at least *N* = 20 was achieved. We recruited 29 research participants, which resulted in a response rate of 72% (*N* = 21); among recruits, one did not respond to recruitment emails and seven declined.

### Measures: Delphi surveys

Participants completed two rounds of feedback using anonymous Delphi surveys. Each survey started with operational definitions of implementation strategies, MBC, school mental health providers, and three vignettes illustrating MBC use in schools (see Supplemental file 1). Vignettes were developed and revised for clarity and accuracy based on feedback from several co-authors and other collaborators. The vignettes focus on MBC clinical practice representing various school mental health professional roles, presenting concerns, student ages^1^, and measures. Due to our focus on identifying implementation strategies for MBC as a clinical practice, the vignettes did not refer to any implementation supports, such as decision support by a measurement feedback system or other digital interface for scoring and viewing progress data. Although clinical decision support tools have been associated with more robust effects of MBC, they are not necessary [56], and using technology to aid measure completion and review may create disparities in MBC access [57]. Availability and feasibility of technology-assisted decision support tools is variable in public schools given the ongoing digital divide in education [58]. Therefore, to ensure MBC was presented in a manner that would not raise resource or equity issues, our vignettes focused on the core components of MBC only, without noting how measures are collected.

The Delphi technique is an established method using a series of surveys or questionnaires to obtain controlled, mixed methods input from a diverse set of expert stakeholders to gain reliable consensus on a health care quality topic [59, 60]. This method was used in the Expert Recommendations for Implementing Change (ERIC) project to identify a complete list of implementation strategies and definitions for selection and application to specific practices and settings [9, 53]. Another research team replicated and extended this research to select and tailor strategies for implementing EBPs in schools [13, 61, 62]. As the prior school study was not practice-specific, we included the 33 implementation strategies rated most important and feasible by the prior study to further refine a list of strategies for MBC in schools [61]. For each strategy, participants indicated whether it is relevant to MBC care specifically (“yes,” “yes with changes,” or “no”). For strategies rated as relevant (“yes” or “yes with changes”), participants then were asked to provide (1) importance and feasibility ratings (1 = “not at all important/feasible” to 5 = “extremely important/feasible”) based on the definition provided, (2) possible synonyms or related activities to the strategy, and (3) suggestions about the definition or application of the strategy. To close the survey, participants were also asked to suggest additional implementation strategies not listed. The Round 2 survey included an updated list of strategies and definitions based on Round 1 results. Participants had 4 weeks to complete Round 1 and 2 surveys. Participants were compensated for their time and study procedures were approved by the Yale Institutional Review Board.

### Data analyses

Descriptive statistics of quantitative feasibility and importance ratings were examined for normality. Independent samples *t*-tests were used to compare ratings between providers and researchers. Mean feasibility and importance ratings were plotted for each strategy on a “go-zone” plot to compare relative feasibility and importance by quadrants [63]. Go-zone plots provide a bivariate display of mean ratings and are often used in concept mapping. The origin represents the grand mean of both variables of interest (in this case, feasibility and importance) and the four resulting quadrants are used to interpret relative distance among items (in this case, strategies). The top right quadrant, Zone 1, is the “go-zone” where strategies of the highest feasibility and importance appear.

A multimethod approach was used to reduce strategies and refine definitions between Survey 1 and Survey 2. First, a document was developed to display quantitative and qualitative Survey 1 results for each strategy. This included each Survey 1 strategy and definition, go-zone quadrant results (overall, as well as for providers and researchers), quantitative considerations (e.g., percentage of stakeholders who indicated the strategy was not relevant for MBC in school, significant differences between providers and researchers, any distribution normality concerns with ratings), qualitative synonyms, and qualitative definition change recommendations made by participants. Second, one rater (EC) reviewed each strategy using this document and established decision-making guidance vetted by study team members for each zone. She coded an initial decision (e.g., retain with revisions, collapse, or remove) with justification for each, documented any synonyms reported more than three times, and drafted definition changes that were (a) minimal language adjustments; (b) not substantial additions to definition length, and (c) consistent with overall scope of the strategy. Then, another rater (CS) reviewed coded decisions and documentation, and all discrepancies were resolved through consensus conversations. Final decisions about collapsing strategies were made based on consultation with two implementation researchers.

We also examined additional strategies and associated definitions recommended by *N* = 10 providers and *N* = 7 researchers as well as substantive comments provided at the end of Survey 1 by *N* = 16 providers and *N* = 8 researchers that pertained to additional strategies. Using thematic analysis and consensus coding by both coders, these data resulted in four distinct strategies broadly related to incentives, policy change, workload/time, and measure selection which were added to Survey 2. We discovered that two strategies (“alter and provide individual- and system-level incentives” and “develop local policy that supports implementation”) already existed in the established list of strategies for EBPs in schools, so we added those strategies and definitions from the published literature [27]. Two strategies (“support workflow adjustments” and “offer a clinician-informed menu of free, brief measures”) were new, so we added those strategies and definitions based on stakeholder qualitative feedback.

To analyze Survey 2 results, descriptive statistics, independent samples *t*-tests and go-zone plots were used again, as was the multi-step process detailed above.

## Results

### Survey 1 strategy ratings

In general, strategies were rated as “relevant” or “relevant with changes” by participants, and all 33 strategies in Survey 1 received importance and feasibility ratings. Eight strategies received the highest proportion of “not relevant” ratings (range = 25–38% participants) to MBC in schools, as follows: (1) model and simulate change; (2) change/alter environment; (3) provide practice-specific feedback; (4) identify early adopters; (5) visit other sites; (6) obtain and use student and family feedback; (7) develop academic partnerships; and (8) build partnerships (i.e., coalitions) to support implementation. Since the majority of participants rated these as “relevant” or “relevant with changes,” the importance and feasibility ratings are included in our analysis.

**Fig. 1:**
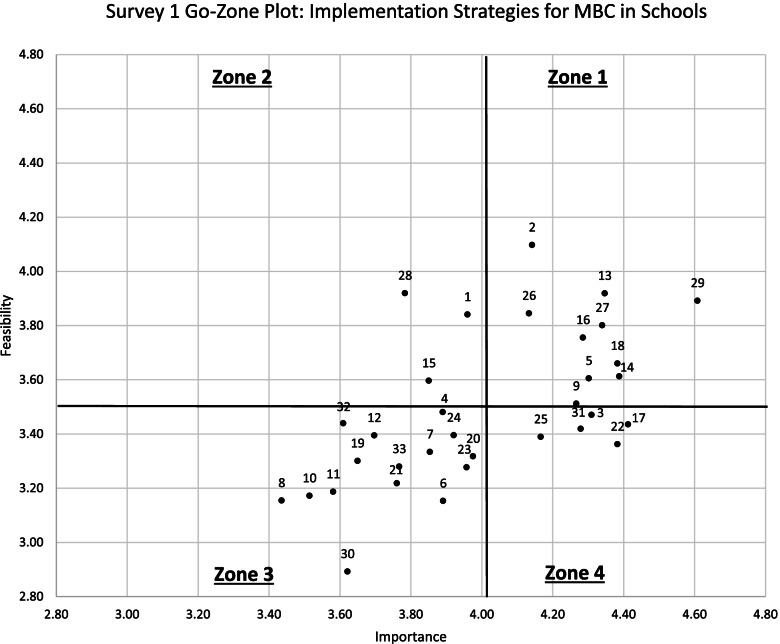
Go-zone plot: Survey 1 importance and feasibility ratings (limited range to focus on origin)

**Fig. 2:**
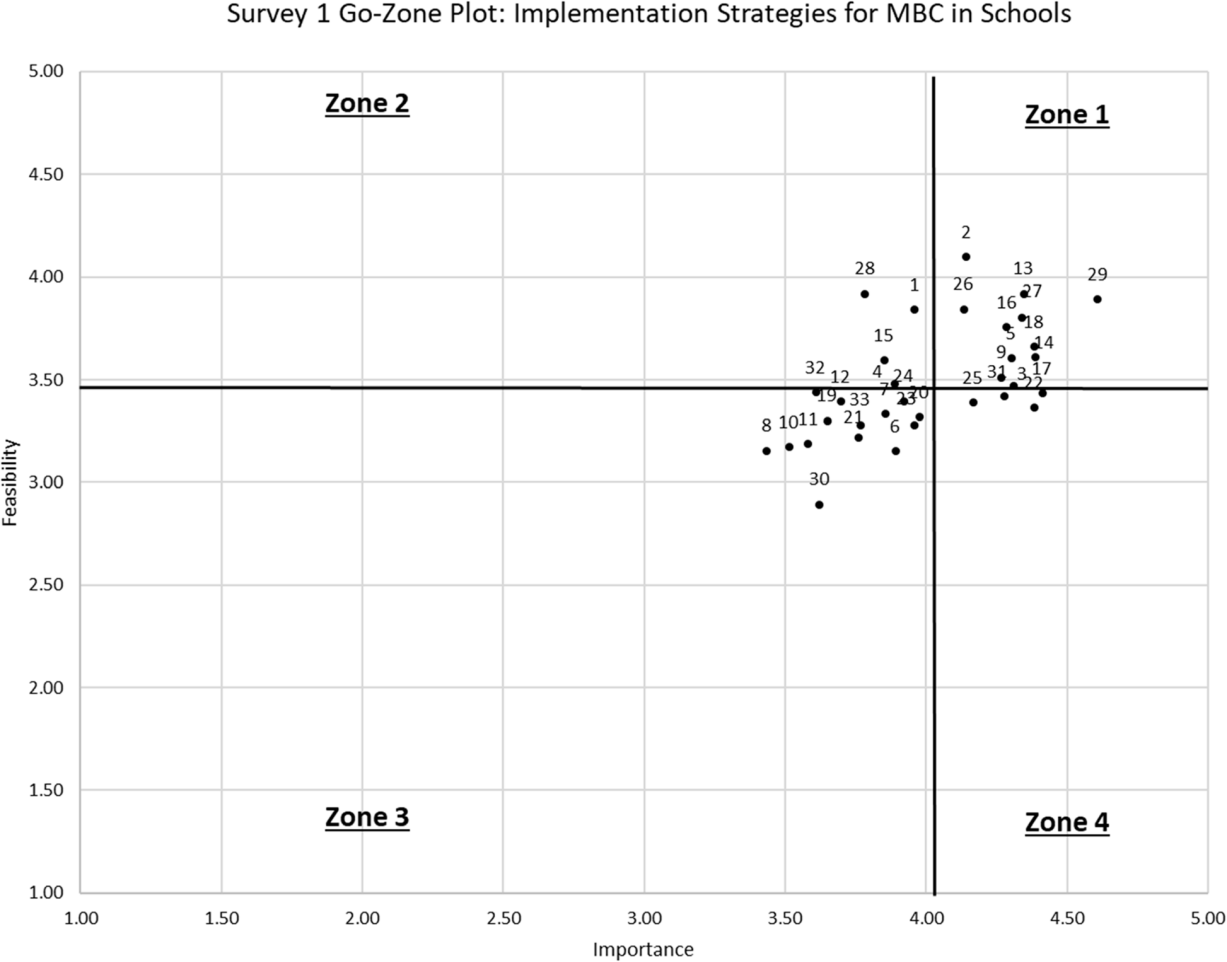
Go-zone plot: Survey 1 importance and feasibility ratings (full range 1–5)

Importance and feasibility ratings were high overall for both survey rounds, with importance ratings higher than feasibility ratings on average. On Survey 1, importance ratings ranged from 3.44 (“develop academic partnerships”) to 4.61 (“make implementation easier by removing burdensome documentation tasks”) and feasibility ratings ranged from 2.89 (“visit other sites”) to 4.10 (“distribute educational materials”). Survey 1 standard deviations varied from 0.68 to 1.18. See Table 2 for importance and feasibility results for the 33 initial implementation strategies. Figures 1 and 2 display these findings on go-zone plots, where the four quadrants or “zones” are divided by the grand mean scores of 4.01 for importance and 3.49 for feasibility. Zone 1 includes strategies rated above the grand mean for importance and feasibility (i.e., high feasibility/high importance), Zone 2 includes strategies rated above the grand mean for feasibility but not importance (i.e., high feasibility/low importance), Zone 3 includes strategies rated below the grand mean for feasibility and importance (i.e., low feasibility/low importance), and Zone 4 includes strategies rated above the grand mean for importance but below the feasibility grand mean (i.e., low feasibility/high importance).

**Table 2:** Results of 33 initial implementation strategies in Survey 1

#	Strategy	Importance				Feasibility				^a^Go-zone	Survey 2 decision
		*N*	*M*	SD	Range	*N*	*M*	SD	Range		
14	Conduct ongoing training	49	4.39	0.84	2.0–5.0	49	3.61	0.95	2.0–5.0	1	Retained
27	Develop implementation plan	50	4.34	0.80	2.0–5.0	50	3.80	0.97	1.0–5.0	1	Retained
9	Develop instruments to monitor and evaluate core components of the innovation/ new practice	45	4.27	0.81	2.0–5.0	45	3.51	0.87	1.0–5.0	1	Retained
2	Distribute educational materials	50	4.14	0.88	2.0–5.0	50	4.10	0.79	2.0–5.0	1	Collapsed w/ 1
16	Facilitation/problem-solving	49	4.29	0.91	1.0–5.0	49	3.76	0.90	2.0–5.0	1	Retained
5	Identify and prepare champions	43	4.30	0.74	3.0–5.0	43	3.60	0.93	2.0–5.0	1	Retained
29	Make implementation easier by removing burdensome documentation tasks	46	4.61	0.68	3.0–5.0	46	3.89	1.18	1.0–5.0	1	Retained
13	Make training dynamic	49	4.35	0.75	2.0–5.0	49	3.92	0.81	2.0–5.0	1	Retained
26	Promote adaptability	45	4.13	0.92	2.0–5.0	45	3.84	0.93	2.0–5.0	1	Retained
18	Provide ongoing consultation/coaching	47	4.38	0.85	1.0–5.0	47	3.66	1.07	1.0–5.0	1	Retained
1	Develop educational materials	50	3.96	0.83	2.0–5.0	50	3.84	0.91	1.0–5.0	2	Collapsed w/ 2
28	Identify early adopters	37	3.78	1.13	1.0–5.0	37	3.92	0.98	2.0–5.0	2	Retained
15	Use train the trainer strategies	47	3.85	1.04	1.0–5.0	47	3.60	0.88	2.0–5.0	2	Retained
23	Audit and provide feedback	47	3.96	0.93	2.0–5.0	47	3.28	0.77	2.0–5.0	3	Collapsed w/ 17&22
19	Build partnerships (i.e., coalitions) to support implementation	40	3.65	0.95	2.0–5.0	40	3.30	1.02	2.0–5.0	3	Collapsed w/ 21
32	Capture and share local knowledge	41	3.61	1.02	2.0–5.0	41	3.44	0.92	2.0–5.0	3	Removed
10	Change/alter environment	35	3.51	1.07	1.0–5.0	35	3.17	1.04	2.0–5.0	3	Removed
33	Conduct education meetings with specific stakeholders	43	3.77	1.02	2.0–5.0	43	3.28	0.98	2.0–5.0	3	Removed
7	Conduct education outreach visits	48	3.85	0.95	1.0–5.0	48	3.33	0.93	2.0–5.0	3	Removed
4	Conduct local needs assessment	46	3.89	1.10	1.0–5.0	46	3.48	0.86	2.0–5.0	3	Removed
21	Create a professional learning collaborative	46	3.76	0.97	1.0–5.0	46	3.22	0.99	2.0–5.0	3	Retained
8	Develop academic partnerships	39	3.44	1.00	1.0–5.0	39	3.15	0.90	2.0–5.0	3	Removed
11	Develop resource sharing agreements	43	3.58	1.20	1.0–5.0	43	3.19	1.01	2.0–5.0	3	Removed
6	Intervene/communicate with students, families, and other staff to enhance uptake and fidelity	46	3.89	1.06	1.0–5.0	46	3.15	0.94	2.0–5.0	3	Removed
20	Involve students, family members, and other staff	41	3.98	1.01	2.0–5.0	41	3.32	1.01	2.0–5.0	3	Retained
12	Model and simulate change	33	3.70	1.05	1.0–5.0	33	3.39	0.97	1.0–5.0	3	Removed
24	Obtain and use student and family feedback	38	3.92	0.88	2.0–5.0	38	3.39	1.03	2.0–5.0	3	Removed
30	Visit other sites	37	3.62	1.14	2.0–5.0	37	2.89	0.97	2.0–5.0	3	Removed
3	Assess for readiness and identify barriers and facilitators	49	4.31	0.82	2.0–5.0	49	3.47	0.96	2.0–5.0	4	Retained
22	Develop a quality monitoring system	47	4.38	0.74	3.0–5.0	47	3.36	0.92	2.0–5.0	4	Collapsed w/ 17 & 23
31	Facilitate relay of intervention and student data to school personnel	43	4.28	0.80	2.0–5.0	43	3.42	0.85	2.0–5.0	4	Removed
17	Monitor the progress of the implementation effort	46	4.41	0.69	3.0–5.0	46	3.43	0.94	2.0–5.0	4	Collapsed w/ 22 & 23
25	Provide practice-specific supervision	36	4.17	0.91	2.0–5.0	36	3.39	0.99	2.0–5.0	4	Retained

*Note*. All retained strategies appeared in Survey 2 with revised strategy names and/or definitions

^a^Go-zone 1 = high feasibility/high importance, go-zone 2 = high feasibility/low importance, go-zone 3 = low feasibility/low importance, go-zone 4 = low feasibility/high importance

### Survey 2 strategy ratings

**Fig. 3:**
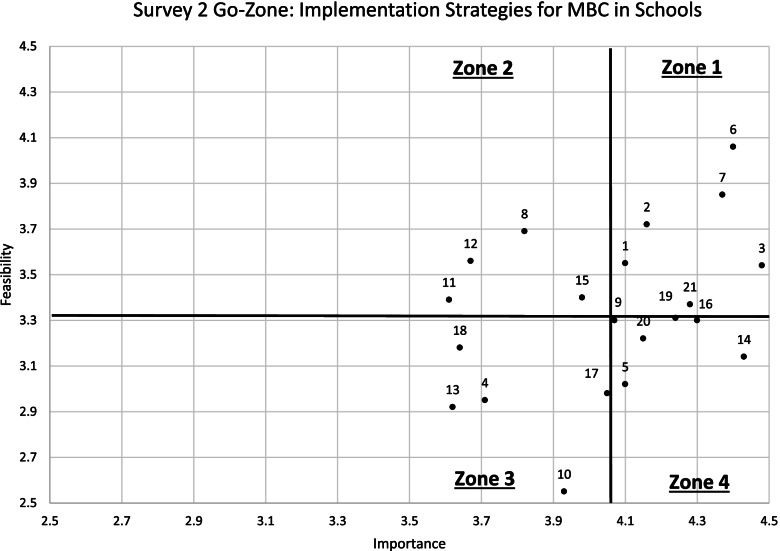
Go-zone plot: Survey 2 importance and feasibility ratings (limited range to focus on origin)

Based on the multimethod approach described above, Survey 2 contained a reduced set of 21 strategies with updated definitions (see Fig. 3). From Survey 1 to Survey 2, 14 strategies were retained (with updates to strategy title and/or definition in most cases), 7 strategies were collapsed into three, 12 were removed, and 4 were added. Feasibility and importance grand means were similar for Survey 2 (importance grand mean = 4.05; feasibility grand mean = 3.33). On Survey 2, importance ratings ranged from 3.61 (“use train the trainer strategies”) to 4.48 (“develop a useable implementation plan”) and feasibility ratings ranged from 2.55 (“support workflow adjustments”) to 4.06 (“offer a provider-informed menu of free, brief measures”). Survey 2 standard deviations varied from 0.56 to 1.22.

### Survey 2 top-rated strategies

**Fig. 4:**
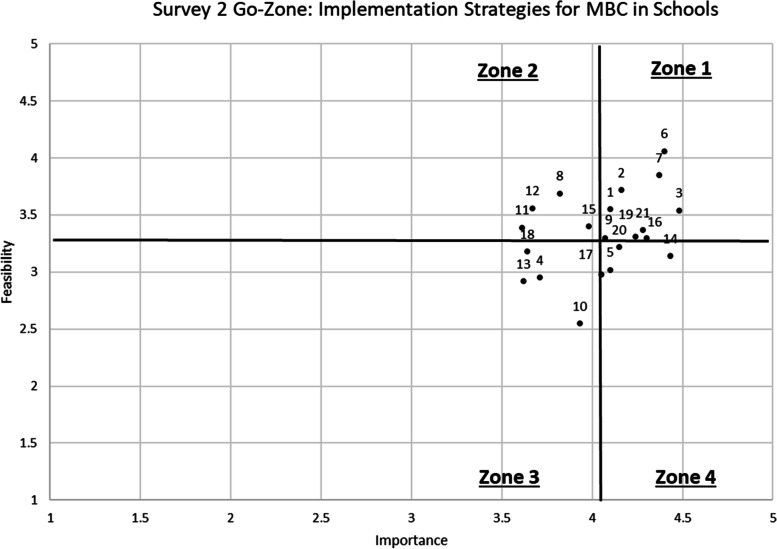
Go-zone plot: Survey 2 importance and feasibility ratings (full range 1–5)

Among the 21 revised implementation strategies included in Survey 2 (see Table 4), six were rated as most important and most feasible (see Zone 1 strategies in Table 3, Fig. 3, and Fig. 4). These top-rated strategies include (1) assess for readiness and identify barriers and facilitators; (2) identify and prepare champions; (3) develop a usable implementation plan; (4) offer a provider-informed menu of free, brief measures; (5) develop and provide access to training materials; and (6) make implementation easier by removing burdensome documentation tasks.

**Table 3:** Results of 21 implementation strategies in Survey 2

#	Strategy	Importance			Feasibility			Go-zone
		*N*	*M*	SD	*N*	*M*	SD	
1	Assess for readiness and identify barriers and facilitators	49	4.10	0.68	49	3.55	0.89	1
2	Identify and prepare champions	43	4.16	0.84	43	3.72	0.77	1
3	Develop a usable implementation plan	48	4.48	0.80	48	3.54	0.90	1
6	Offer a provider-informed menu of free, brief measures	48	4.40	0.79	48	4.06	1.02	1
7	Develop and provide access to training materials	48	4.38	0.84	48	3.85	0.92	1
21	Make implementation easier by removing burdensome documentation tasks	46	4.28	0.94	46	3.37	1.18	1
8	Make training dynamic	45	3.82	0.98	45	3.69	0.97	2
11	Use train the trainer strategies	44	3.61	1.13	44	3.39	1.04	2
12	Identify early adopters	39	3.67	0.87	39	3.56	0.91	2
15	Provide practice-specific supervision	40	3.98	1.00	40	3.40	1.11	2
4	Alter and provide individual- and system-level incentives	38	3.71	0.98	38	2.95	0.87	3
10	Support workflow adjustments	40	3.93	0.83	40	2.55	0.85	3
13	Facilitation	37	3.62	1.06	37	2.92	0.86	3
17	Involve students, family members, and other staff	42	4.05	0.91	42	2.98	1.09	3
18	Create a professional learning collaborative	44	3.64	0.97	44	3.18	1.06	3
5	Develop local policy that supports implementation	42	4.10	0.93	42	3.02	1.00	4
9	Conduct ongoing training^a^	43	4.07	0.83	43	3.30	0.91	4
14	Provide ongoing clinical consultation/coaching^a^	44	4.43	0.73	44	3.14	0.98	4
16	Monitor implementation progress and provide feedback^a^	44	4.30	0.70	44	3.30	0.95	4
19	Monitor fidelity to MBC core components^a^	42	4.24	0.69	42	3.31	0.81	4
20	Promote adaptability^a^	40	4.15	0.98	40	3.23	0.80	4

^a^These strategies were less than 0.50 of the mean cutoffs for feasibility, yet above the mean cutoff for importance

Several additional strategies were rated within 0.50 of the feasibility grand mean, yet above the mean cutoff for importance (see Table 3, Zone 4 strategies with asterisks). These include “conduct ongoing training,” “provide ongoing clinical consultation/coaching,” “monitor implementation progress and provide feedback,” “monitor fidelity to MBC core components,” and “promote adaptability”.

### Stakeholder group comparisons

On Survey 1, provider and researcher ratings were not significantly different with three exceptions. First, as compared to researchers, providers reported that it is more feasible and important to make implementation easier by removing burdensome paperwork (feasibility provider *M* = 4.31 vs researcher *M* = 3.35; feasibility *t* [44] = -2.96, *p* = 0.01, *d* = 0.88; importance provider *M* = 4.85 vs researcher *M* = 4.30; importance *t* [44] = 2.90, *p* < 0.01, *d* = 0.86). Second, as compared to providers, researchers reported it is more important to monitor the implementation effort (provider *M* = 4.20 vs researcher *M* = 4.67; *t* [44] = −2.51, *p* = 0.02, *d* = −0.72). Third, train-the-trainer feasibility ratings were significantly higher among providers (*M* = 3.81) than researchers (*M* = 3.30; *t* [45] = 2.06, *p* < 0.05, *d* = 0.61). On Survey 2, provider and researcher ratings were not significantly different with one exception; providers reported it is more important to make implementation easier by removing burdensome paperwork (provider M = 4.50 vs researcher *M* = 3.94; *t* [44] = 2.04, *p* = 0.048, *d* = 0.62).

## Discussion

We applied an established, stakeholder-informed method to identify important and feasible implementation strategies for measurement-based care (MBC) used in school-based mental health treatment. MBC was selected as an under-implemented yet promising and scalable clinical practice in schools that can be added to any presenting concern or treatment plan to improve care quality for children and adolescents. We identified six top-rated implementation strategies for MBC based on ratings of importance and feasibility in schools. Those strategies were (1) assess for readiness and identify barriers and facilitators; (2) identify and prepare champions; (3) develop a usable implementation plan; (4) offer a provider-informed menu of free, brief measures; (5) develop and provide access to training materials; and (6) make implementation easier by removing burdensome documentation tasks.

These six strategies identified represent a natural chronology for organizing an implementation approach for clinical providers in schools. For example, several strategies could be put in place before an initial training or provision of training materials occurs (e.g., assess for readiness, develop an implementation plan) and others could follow. These strategies could also be provided as a “bundle” to support MBC implementation in schools.

Several additional strategies were rated as highly important and relatively feasible within 0.50 of the feasibility grand mean. In general, these strategies reflect those that promote ongoing implementation in clinical practice after initial planning and provider training, which is highly consistent with extant findings about the importance of post-training implementation support strategies [64–66]. As these strategies are near the “border” of feasibility and importance grand means, they warrant attention as potentially viable strategies, given the strictly numeric, bivariate cutoff between zones based on grand mean values.

Implementation and feasibility ratings were not significantly different between providers and researchers, although future replication with a larger sample size is warranted. The few significant differences identified involved moderate to large effect sizes, with providers emphasizing the reduction of burdensome documentation and researchers emphasizing fidelity monitoring to support MBC in schools. These differences have face validity; providers have more experience with barriers related to documentation and other clinical workflow details than researchers do, and researchers are more focused on ensuring the implementation is carried out as intended. These differences illustrate the importance of ensuring bidirectional communication, collaboration, and perspective sharing between these two groups of stakeholders, and highlight the importance of sampling various stakeholder perspectives when examining implementation processes.

Also, by focusing specifically on MBC implementation in schools, the current results reveal a narrower and a higher range of both importance and feasibility ratings for MBC implementation strategies in schools as compared to general EBP implementation (our importance range = 3.61–4.48 versus a range of 2.62–4.59 in prior work and our feasibility range = 2.55–4.06 versus a range of 2.08–3.72 in prior work [54]. These differences suggest the value of prioritizing implementation strategies to specific implementation settings and contexts as was the case in this study.

## Limitations

This study has several limitations. First, although this sample was nationally representative, it is relatively small, and thus importance and feasibility ratings may not hold for a larger sample. Degrees of freedom were further limited by only requesting feasibility or importance ratings if the participant responded that the strategy was relevant to MBC. Also, school providers were recruited from a national dataset of teams engaged in school mental health quality assessment and improvement efforts, which may be a more select group of school mental health providers. Future studies should examine importance and feasibility ratings from a wider range of school mental health providers. A larger sample would also allow for more powered analyses of school and provider characteristics (e.g., school size, provider characteristics in Table 1) as moderators of feasibility and importance ratings.

Also, we selected 33 implementation strategies already rated highly in a prior study of EBP implementation in schools, and thus we were unlikely to find mean importance or feasibility ratings in the low to moderate range. Although this may raise questions about potential ceiling effects, the grand means for each construct were not overly high (importance grand mean = 4.05; feasibility grand mean = 3.33), and we used the grand mean as the cut point for the sample (as is conventional for go-zone graphs) to interpret differences among ratings.

Finally, stakeholders’ qualitative feedback about the definition of each strategy was used to develop the final list that appears in Table 4, but recommendations about application of the strategy were not included. This is most pertinent to feasibility, and our team is currently examining these qualitative data to understand how we might optimize feasibility of individual strategies that were rated highly important, but less feasible (Awad M, Connors E: Promoting measurement-based care in school mental health through practice-specific supervision, submitted). Feasibility is a complex construct; many elements contribute to feasibility ratings for a given practice or strategy [67] and when we assess perceptions of feasibility prospectively, the rater has to make assumptions about what resource or training requirements, for example, are part of the strategy [7]. It is not uncommon for school stakeholders to rate implementation supports or best practices as more important than feasible due to their experience with resource constraints and structural barriers in schools [16, 68]. Therefore, future research should continue to examine how to operationalize, tailor, and evaluate strategies to promote feasibility in the school context, in order to support schools’ capacity to feasibly implement new initiatives with integrity and sustainability [33, 69].

**Table 4:** Final list of 21 implementation strategies and definitions for MBC in school mental health

Strategy	Definition
1. Assess for readiness and identify barriers and facilitators^d^	Assess readiness for MBC at provider, administration, and school setting levels. Identify strengths or facilitators that can support the implementation effort and barriers that might get in the way. This could occur before, during, and/or after implementation with providers, primarily, as well as other stakeholder groups.
2. Identify and prepare champions^d^	Identify individuals who are passionate about MBC in schools and are influencers or informal leaders among fellow providers. Prepare and support them to facilitate implementation, support their peers, and overcome or address indifference or resistance that MBC may provoke in a school or district. There may be more than one champion per school site. Sites may have the option to adjust this title to local language (e.g., MBC key opinion leader, lead provider, coach, intervention specialist).
3. Develop a usable implementation plan^d^	Develop a usable plan for implementation built around student outcomes as the ultimate goal of MBC implementation effort. The implementation plan will detail processes and strategies that will be used to achieve those goals. The plan should also include timeframe and milestones, roles and responsibilities of all stakeholders, and appropriate performance/progress measures. Use and update this plan to guide the implementation effort over time. It will be used to promote excitement and buy-in, collaborative planning, clear communication and training and adaptive implementation over time.
4. Alter and provide individual- and system-level incentives^d^	Work to provide individual-level (e.g., provider recognition, acknowledgement, gift card) and system-level (e.g., grant money, free training and consultative support) incentives to districts or schools to participate and engage in an MBC implementation effort.
5. Develop local policy that supports implementation^b^	Develop local school system policy that establishes rules, expectations, and guidelines for MBC implementation.
6. Offer a provider-informed menu of free, brief measures^c^	Engage providers in a discussion about measure selection to select and distribute a small number of progress monitoring tools. Emphasize tools that are free, brief, and easy to score.
7. Develop and provide access to training materials^d^	Training materials (i.e., a curriculum, toolkit, or guide) for MH professionals would include what MBC is, why MBC is important, goals of MBC, clear steps to follow, examples and non-examples of proper MBC, implementation scripts, practice profiles, timelines, and rating scales for use. The study team would develop these materials with school provider stakeholder input and would work with schools and mental health agencies to distribute materials to school providers electronically. Materials would be made available to providers following the training.
8. Make training dynamic^a^	Ensure the initial training is interactive, experiential and relevant to providers (e.g., to include role plays, MBC practice examples and non-examples, MBC research base, planning ahead for MBC implementation with students served, and discussion). Make information available in multiple formats. Vary how information is delivered for various professional development schedules and structures.
9. Conduct ongoing training^a^	Plan for and conduct one or more “booster” or follow-up trainings after the initial training. (Note: This is different from ongoing clinical consultation/coaching, or supervision.)
10. Support workflow adjustments^c^	Provide protected time for individualized implementation planning about how the provider can integrate MBC into their existing workflow and problem solve anticipated barriers. This is intended to acknowledge providers’ limited time and provide support for self-reflection and personalized action planning. May involve engaging providers’ supervisor or building administrator for support. This could occur at the initial training, booster training, or during ongoing consultation.
11. Use train the trainer strategies^a^	Train designated, local school providers to train other school mental health providers in MBC using a systematic process to support ongoing implementation and sustainability.
12. Identify early adopters^a^	Identify early adopters (i.e., individuals who are particularly open to change) of MBC within the school, district or community agency to learn from their experiences and demonstrate the benefit of MBC to other providers. Early adopters could share their experiences of success with others and be involved in ongoing training and consultation efforts if they are willing. This strategy is used after implementation has started for everyone; it is different than piloting with a small group of enthusiastic providers first before implementing.
13. Facilitation^d^	A process of interactive implementation support that is provided by an internal or external facilitator to the whole school or district system. Facilitation should be non-evaluative, informative and part of a supportive interpersonal relationship. Usually provided by someone who works with school leaders, providers, and all other stakeholders to problem solve and tailor the types of support provided based on specific barriers or challenges with MBC implementation. For example, a facilitator could help address systemic barriers to implementation based on what stakeholders report and recommend. This is different from ongoing clinical coaching or consultation to providers to help them implement MBC with their students directly.
14. Provide ongoing clinical consultation/ coaching^a^	Provide ongoing clinical consultation and coaching by one or more experts or trained clinical peers. Consultation and coaching would be non-evaluative, flexible, individualized, and focused on helping providers improve their MBC implementation. This includes problem solving and performance feedback throughout implementation. (NOTE: Clinical consultation / coaching is typically differentiated from the next strategy, supervision, which is usually provided by an internal individual who has supervisory authority over the implementer.)
15. Provide practice-specific supervision^b^	Provide school providers with supervision focusing on MBC. Supervisors are in a position of authority and support school providers who deliver new practices with evaluative feedback via performance assessment. (Note: Supervision is typically differentiated from consultation/ coaching, which may be provided by an internal or external individual who may or may not have authority over the implementer.)
16. Monitor implementation progress and provide feedback^d^	Collect and summarize data regarding MBC implementation outcomes (fidelity, acceptability, how many providers are using it) over a specified time period and give it to administrators, school personnel and/or providers to monitor, evaluate, and support providers’ MBC practices. The purpose of this strategy is to continuously improve the quality of implementation and inform data-driven, real-time decisions about what supports providers need most. To do this, a quality monitoring system and procedures would first need to be developed. Also referred to as “audit and provide feedback”. (Note: This is included in strategies 13. Facilitation and 14. Ongoing clinical consultation/coaching.)
17. Involve students, family members, and other staff^a^	Ask students, family members, and providers, as those receiving and providing MBC, to provide input and recommendations about implementation to improve practice and quality. Topics may include how school providers can most effectively implement MBC (to collect, share, and act on student progress data), how to ensure students and families can be actively involved in MBC, ways to make MBC purpose clear to everyone, ensuring an equal student-parent-provider partnership, addressing concerns or barriers, and/or what implementation supports are needed.
18. Create a professional learning collaborative^a^	Facilitate the formation of school provider groups within or between school systems or mental health agencies to foster a collaborative learning environment to improve MBC implementation. Providers could network with others in their district or beyond who are also implementing MBC to share resources, lessons learned, and support with the help of a learning collaborative facilitator. The learning community would be organized, developed and coordinated by a research team or implementation consultant (not the providers).
19. Monitor fidelity to MBC core components^d^	Integrate measurement strategies to assess the degree to which MBC core components (i.e., collecting, sharing, and acting on the student progress data) is occurring during implementation. For example, study team or clinical supervisors could review IEPs, 504 plans or treatment plans for documentation of MBC. The purpose is to inform ongoing quality improvement and implementation supports.
20. Promote adaptability^a^	Identify ways MBC can be tailored or adapted to best fit with the school/classroom context, meet local needs (e.g., selecting measures most appropriate for student characteristics, cultural and linguistic competencies) and clarify which elements of MBC must be maintained to preserve fidelity. The MBC implementation study team and school personnel (mental health providers, administrators) would work together on adaptations or tailoring needed to improve feasibility, acceptability, and appropriateness of MBC. Adaptations should be documented and based on provider, student and/or family feedback after initial implementation with fidelity.
21. Make implementation easier by removing burdensome documentation tasks^a^	Remove or alleviate burdensome tasks or documentation that could come with implementing MBC (e.g., removing unnecessary or unused data forms, streamlining duplicative paperwork, require only minimal necessary documentation, and make sure all data collected are used). This should apply to measures collected (i.e., improve efficiency with user-friendly forms and auto scoring) and progress note documentation (i.e., templates to document MBC data results and how they were used in session with the student and family).

^a^These strategies reflect the original strategy name and definition from Lyon et al. [62], with additional language or information specific to MBC implementation based on stakeholder feedback

^b^These strategies reflect the original strategy name and definition from Lyon et al. [62], with very minor adjustments (i.e., “MBC implementation” vs “new practices”)

^c^ These strategies were generated through this study

^d^ These strategies originate from Lyon et al. [62] but the strategy name and/or definition is altered greatly and/or collapsed with other strategies, based on stakeholder feedback regarding MBC implementation strategies in schools

## Conclusion and future directions

Methods to select and tailor implementation strategies for a particular practice and setting have been somewhat elusive to date in implementation research and practice [5]. The methods used in this study can be applied to other evidence-based practices, settings, and contexts to solve implementation challenges. In addition, the effectiveness of implementation strategies selected for their potential importance and feasibility needs to be empirically examined. Identification of top-rated strategies for a particular intervention and context is foundational to future strategy testing with practicing providers in real-world care systems. Strategies selected from implementation science methods, such as the current survey methods with go-zone plots, should also be critically examined for the possibility of bundling or combining some strategies together (for parsimony and/or alignment) as well as when to apply strategies across implementation stages over time.

## Supplementary Information

**Additional file 1.**

## Data Availability

The datasets generated during and/or analyzed during the current study are not publicly available due to the pilot nature of the study and lack of patient outcome data but are available from the corresponding author on reasonable request.

## Abbreviations

MBC: Measurement-Based Care
EBP: Evidence-based practice

## Footnotes

1 Vignettes refer to student “grade”, not age. In the United States, 3^rd^ grade is in primary school, approximately 8 years old, 6^th^ grade is considered “middle school”, approximately 11 years old, and 9^th^ grade is the beginning of secondary school, approximately 14 years old.
