# Supplementary material for: Implementation strategies to promote measurement-based care in schools: evidence from mental health experts across the USA
Source: Implement Sci Commun. 2022 Jun 21;3:67. doi: 10.1186/s43058-022-00319-w (PMC9210728; doi:10.1186/s43058-022-00319-w)
Supplement: Supplementary file 1 — Additional file 1. [file 43058_2022_319_MOESM1_ESM.pdf]

Using Data in School Mental Health: Definitions and Vignettes

Implementation Strategies: An activity or set of activities to facilitate adoption of new practices into routine care, usually within a healthcare or related service delivery system. These activities include planning (e.g., building buy-in and relationships), education (e.g., training, communications), financing (e.g., incentives and resources), quality management (e.g., fidelity monitoring, useful data systems) and policy (e.g., changes with licensing boards, regulations or accrediting bodies). In this case, implementation strategies are what we DO to help clinicians collect and use more data when providing mental health treatment in schools.

School mental health clinicians: Any school-based mental health professional who provides mental health treatment to students and their families. Clinicians may be school-employed (e.g., school social worker, school psychologist, school counselor) or community-employed (e.g., social worker, psychologist, marriage and family therapist, psychiatrist) as long as they provide treatment services from a location on the school campus. Note: School health providers (e.g., school nurses) often provide direct mental health care in schools but are NOT included for the purposes of this project because their role with mental health is slightly different.

Measurement-based care (MBC): The routine collection and use of patient-reported data throughout treatment. Brief, valid and reliable student- or parent- reported symptom and functioning data are collected and used during 1) initial screening and assessment, 2) problem definition and analysis, 3) finalizing treatment goals and intervention tactics, 4) monitoring treatment progress and 5) ongoing feedback and collaboration with the student and their family. The goal of using MBC practices is to drive collaborative decision-making with the student and family throughout the treatment process, including when to keep interventions the same, when to make a change, and when it's time to end treatment due to success in reaching the identified goals. The clinician selects the measure or set of measures used based on the individual student. Additional description of MBC and its benefits can be found at https://www.mirah.com/mbc

NCSMH NATIONAL CENTER FOR SCHOOL MENTAL HEALTH

This study is a partnership between the Division of Prevention and Community Research at the Yale School of Medicine and the National Center for School Mental Health at the University of Maryland School of Medicine.

UNIVERSITY of MARYLAND SCHOOL OF MEDICINE

Yale SCHOOL OF MEDICINE

Sample Vignettes: What MBC "looks like" in schools

Vignette 1: Ms. Sanchez, a school social worker, is working with a Terrance, a 9th grader who has an Individualized Education Program (IEP) to receive mental health counseling for Attention-Deficit/Hyperactivity Disorder – Combined Type. The Vanderbilt Rating Scale was collected from his mother and two teachers during his initial assessment which confirmed his diagnosis of ADHD. After discussing the results, Ms. Sanchez, Terrance and his mother determined that the most pressing concern for Terrance was having an organizational system with built-in reminders to track, complete and turn in his school assignments. They decided to track Terrance's progress by 1) a once-weekly review of his daily agenda and 2) monitoring how many days he turns in his homework to all 5 academic classes. His progress with using the agenda and turning in homework was reviewed at the beginning of each session to discuss how well the different organizational strategies taught by Ms. Sanchez were working for him. Ms. Sanchez also collected the Vanderbilt on a quarterly basis to monitor and discuss with Terrance his overall ADHD symptoms and functioning in school. These data were all used in Terrance's year-end IEP meeting to assess his progress and make changes as necessary to his goals and accommodations.

Vignette 2: Mr. Koi, a licensed professional counselor who works for ABC Family Services, spends three days per week working with students at Oak Elementary/Middle School. Christina is a 3rd grade student at Oak who was referred to Mr. Koi for inattention, frequent late arrivals to school and sometimes falling asleep in class. As part of the intake process, Mr. Koi collected the UCLA-PTSD Index from Christina and her mother and discovered she has Post-Traumatic Stress Disorder (PTSD). Christina's most distressing symptom was nightmares which made it difficult for her to sleep through the night and caused many problems with her attendance and grades. Mr. Koi started an evidence-based trauma treatment, and Christina and her father worked together to track her use of coping skills and her sleep on a weekly basis. Data collection and feedback with Christina's father sometimes needed to occur over the phone due to his work schedule. After six weeks, Christina was coping much better with her trauma symptoms but was still having sleep disruptions, so Mr. Koi started including sleep hygiene components in her treatment. A more consistent bedtime routine turned out to be very helpful for Christine getting to bed on time even after her coping skills improved and nightmares started subsiding. The UCLA-PTSD Index was re-administered six months later to determine whether Christina's trauma symptoms were reduced to sub-clinical levels to successfully end treatment.

Vignette 3: Dr. Black, a clinical psychologist who sees students at Tangier Middle School, is working with Tyler, a 6th grade student who was referred for anger outbursts in school and declining grades. His parents are going through a divorce and reported he is worried about possibly having to move. Tyler was irritable and guarded when he met Dr. Black, so Dr. Black needed to reply on parent and teacher report for initial assessment and treatment goal setting. The Strengths and Difficulties Questionnaire was collected at the beginning and throughout treatment as well as a behavior log for skill use (completed by the teacher) to build on Tyler's strengths and assess and adjust different treatment approaches. Dr. Black also used the four-item Session Rating Scale (SRS) with Tyler to obtain feedback on their working alliance from Tyler's perspective. As Tyler began to trust Dr. Black more and their relationship improved, Tyler became more honest on the SRS score and started to talk about what was on his mind during the school day that resulted in his problematic classroom behaviors.

NCSMH
NATIONAL CENTER FOR SCHOOL MENTAL HEALTH

This study is a partnership between the Division of Prevention and Community Research at the Yale School of Medicine and the National Center for School Mental Health at the University of Maryland School of Medicine.

UNIVERSITY of MARYLAND SCHOOL OF MEDICINE

Yale SCHOOL OF MEDICINE
